# Learning From the Experiences of COVID-19 Survivors: Web-Based Survey Study

**DOI:** 10.2196/23009

**Published:** 2021-05-11

**Authors:** Temiloluwa Prioleau

**Affiliations:** 1 Dartmouth College Hanover, NH United States

**Keywords:** patient-reported outcomes, coronavirus, COVID-19, outcome, crowdsourcing, social media, internet, survivor, experience

## Abstract

**Background:**

There are still many unanswered questions about the novel coronavirus; however, a largely underutilized source of knowledge is the millions of people who have recovered after contracting the virus. This includes a majority of undocumented cases of COVID-19, which were classified as mild or moderate and received little to no clinical care during the course of illness.

**Objective:**

This study aims to document and glean insights from the experiences of individuals with a first-hand experience in dealing with COVID-19, especially the so-called mild-to-moderate cases that self-resolved while in isolation.

**Methods:**

This web-based survey study called C19 Insider Scoop recruited adult participants aged 18 years or older who reside in the United States and had tested positive for COVID-19 or antibodies. Participants were recruited through various methods, including online support groups for COVID-19 survivors, advertisement in local news outlets, as well as through professional and other networks. The main outcomes measured in this study included knowledge of contraction or transmission of the virus, symptoms, and personal experiences on the road to recovery.

**Results:**

A total of 72 participants (female, n=53; male, n=19; age range: 18-73 years; mean age: 41 [SD 14] years) from 22 US states were enrolled in this study. The top known source of how people contracted SARS-CoV-2, the virus known to cause COVID-19, was through a family or household member (26/72, 35%). This was followed by essential workers contracting the virus through the workplace (13/72, 18%). Participants reported up to 27 less-documented symptoms that they experienced during their illness, such as brain or memory fog, palpitations, ear pain or discomfort, and neurological problems. In addition, 47 of 72 (65%) participants reported that their symptoms lasted longer than the commonly cited 2-week period even for mild cases of COVID-19. The mean recovery time of the study participants was 4.5 weeks, and exactly one-half of participants (50%) still experienced lingering symptoms of COVID-19 after an average of 65 days following illness onset. Additionally, 37 (51%) participants reported that they experienced stigma associated with contracting COVID-19.

**Conclusions:**

This study presents preliminary findings suggesting that emphasis on family or household spread of COVID-19 may be lacking and that there is a general underestimation of the recovery time even for mild cases of illness with the virus. Although a larger study is needed to validate these results, it is important to note that as more people experience COVID-19, insights from COVID-19 survivors can enable a more informed public, pave the way for others who may be affected by the virus, and guide further research.

## Introduction

The COVID-19 pandemic has significantly impacted the majority of countries in the world, with a total of more than 41.1 million confirmed cases and over 1.1 million deaths reported worldwide as of October 22, 2020 [1]. Months into the pandemic, there are still many unknowns about the novel coronavirus, its transmission, and the experiences of people who have been infected by the virus [2,3]. However, it is known that the disease severity of COVID-19 can range from mild to critical. Studies from the Centers for Disease Control and Prevention (CDC) in the United States and China show that around 80% of the COVID-19–infected population experience “mild-to-moderate” symptoms [4,5]. Due to the sheer volume of infected persons and the limited capacity of the health care systems, it was and still is recommended that patients with mild-to-moderate cases of COVID-19 manage their illness in isolation [2]. Many of these patients, especially in the early months of the outbreak (ie, February and March 2020 in the United States [6]), received little to no clinical care [7]; hence, there is a critical gap in knowledge about their experiences with COVID-19. According to the CDC, “recognition of factors associated with [the] amplified spread during the early acceleration period will help inform future decisions” and “strengthen systems to detect potential transmission resurgence” [6].

Unlike hospitalized patients, the population of people with mild-to-moderate symptoms of COVID-19 remains largely understudied and are among the “undocumented masses”; yet this population contributes significantly to the rapid transmission of the virus [3,8,9]. To fill in the gap, this study aims to enable the research and larger community to glean insights from the experiences of COVID-19 survivors, especially those whose journey with the virus is not captured in clinical or medical records. Through personal stories, this study aims to bring awareness to how people in the United States contracted the coronavirus, the range of symptoms experienced during illness, duration of illness, and their experiences on the road to recovery. Such knowledge can inform guidelines for the vast majority of cases of COVID-19 that are considered to be of mild-to-moderate severity, especially given the current resurgence in the United States [10]. In this work, we developed and deployed a web-based platform for collating experiential data from persons who recovered and/or are on the road to recovery from COVID-19. As previously mentioned, the primary aim of this study is to garner insights from persons with first-hand experience with COVID-19, especially cases of mild-to-moderate severity that are less documented in literature. A total of 72 subjects from 22 US states with laboratory-confirmed positive tests for COVID-19 (68/72, 94%) and presumptively positive as identified by clinical personnel (4/72, 6%) were recruited to share their experience with COVID-19. Details on the recruitment and participant characteristics are described in the Methods section.

## Methods

### Study Description

This research study was approved by Committee for Protection of Human Subjects (CPHS) at Dartmouth College, New Hampshire, United States, and is reported in accordance with the CHERRIES (Checklist for Reporting Results of Internet E-Surveys) statement for web-based surveys [11]. We recruited COVID-19 survivors defined as persons who tested positive for coronavirus and/or who were later confirmed to have had the virus by testing positive for antibodies. Participants were recruited through multiple sources, including online support groups such as Survivor Corps [12], advertisement in local newspapers, and by sharing about the research study through professional and other networks. In total, 72 participants were recruited from May 10 through October 1, 2020, although the majority (ie, 54/72, 73%) of the participants were recruited between May 10 and June 18, 2020. About half of the participants in this study (35/72, 49%) were recruited from the Survivor Corps online support group following the participant’s self-identification of having had COVID-19. For this population, a recruitment message was sent directly to prospective participants informing them about the study and requesting their participation if they were eligible. All interested participants who learned about the study were directed to the project website and invited to first complete a presurvey on the *C19 Insider Scoop* project website to screen for the following eligibility criteria: 18 years or older, living in the United States, and tested positive for COVID-19 and/or COVID-19 antibodies. Participants who met these eligibility requirements were then provided a password by email and a link to the full survey, through which they shared their story of COVID-19. In total, 105 prospective participants completed the presurvey and 72 participants completed the full survey, thereby providing data to be presented in this study. Eligible participants who completed the study requirements (ie, presurvey and full survey) were provided the option of receiving a monetary incentive for participation.

The full survey was developed using the well-established Qualtrics survey software under Dartmouth College’s license to ensure secure data storage behind institutional firewalls. The survey questions in this study were informed by various sources, including the CDC’s coronavirus case report form [13], the National Institutes of Health (NIH) repository of COVID-19 research tools [14], and knowledge gaps identified in the literature [7]. The full survey included questions covering the themes of descriptive characteristics, contraction or transmission of the virus, symptoms and coping strategies, and the road to recovery. In addition, adaptive questioning was implemented such that some questions were only displayed based on responses to previous items. On two occasions, additions were made to the full survey during data collection. More specifically, on May 21, 2020, the symptoms of headaches, muscle or body aches, and dizziness were added to the default checklist provided in this study, after 20 participants had completed the full survey. On May 28, 2020, questions on antibody testing were added, after 32 participants had completed the full survey. Participants who completed the survey before each of these revisions were made have no responses to the new questions added after their participation in the survey. An exact copy of the full survey used in this study is presented in Multimedia Appendix 1.

A complete demographic summary of the participants is presented in Table 1. Of the 72 (female, n=53; male, n=19) participants, 68 (94%) received a positive laboratory test and/or positive antibody test for COVID-19, and the remaining 4 (6%) were confirmed presumptively positive by clinical personnel. Participants’ age ranged from 18 to 73 years. They were residents of a total of 22 US states, with the highest representation from New York. The race demographics included 43 (60%) White, 14 (19%) Black or African American, 6 (8%) Asian, and 2 (3%) American Indian or Alaska Native participants, and the remaining 7 (10%) reported “other” or mixed race. In addition, a total of 23 (32%) participants had at least one pre-existing medical condition, and 6 (8%) participants had more than one pre-existing medical condition. The most common pre-existing conditions reported included asthma (15/72, 21%), high blood pressure (4/72, 6%), and immunosuppressive conditions (3/72, 4%). Other descriptive factors such as the participants’ highest level of education, household income, and occupation are detailed in Table 1.

The following subsections describe other categories of the full survey, the results of which will be presented, including (1) onset, testing, and contraction of COVID-19; (2) a deeper look at COVID-19 symptoms; (3) the road to recovery from COVID-19; and (4) insights from COVID-19 survivors.

**Table 1:** Demographic summary of participants (ie, COVID-19 survivors) in the C19 Insider Scoop Study (N=72).

Characteristics		Value
Age (years), mean (SD)		41 (14)
Age range (years)		18-73
**Sex, n (%)**		
	Female	53 (74)
	Male	19 (26)
**Race, n (%)**		
	White	43 (60)
	Black or African American	14 (19)
	Asian	6 (8)
	American Indian or Alaska Native	2 (3)
	Other or Mixed race	7 (10)
**US state of residence, n (%)**		
	New York	25 (35)
	California	5 (7)
	Georgia	5 (7)
	Massachusetts	4 (6)
	Virginia	4 (6)
	Texas	3 (4)
	New Hampshire	3 (4)
	8 other states ^a^	2 (3)
	7 other states^b^	1 (1)
**Pre-existing medical condition, n (%)**		
	No, with pre-existing condition	48 (68)
	Yes, with pre-existing condition	23 (32)
	Not reported	1 (1)
**COVID-19 testing, n (%)**		
	Positive laboratory or antibody test	68 (94)
	Presumptively positive	4 (6)
**Highest level of education, n (%)**		
	4-year degree	25 (35)
	Professional degree	18 (25)
	Some college	14 (19)
	High school or GED^c^	6 (8)
	Doctorate	5 (7)
	2-year degree	4 (6)
**Household income level (US $), n (%)**		
	<20,000	13 (18)
	40,000-79,999	9 (12)
	80,000-139,999	25 (35)
	140,000-199,999	15 (21)
	≥200,000	9 (12)
	Not reported	1 (1)
**Occupation, n (%)**		
	Nonessential worker	20 (28)
	Essential worker (health care)	14 (19)
	Essential worker (non–health care)	14 (19)
	Unemployed	12 (17)
	Student	7 (10)
	Retired	5 (7)

^a^Alabama, Ohio, Illinois, Maryland, Louisiana, North Carolina, Washington, Vermont

^b^Indiana, Missouri, Arkansas, Wisconsin, New Jersey, Connecticut, Pennsylvania

^c^GED: Tests of General Educational Development.

### Onset, Testing, and Contraction of COVID-19

Within the full survey, there were a total of 8 questions that addressed topics related to the onset, testing, and contraction of COVID-19, including the following:

1. Did you test positive for COVID-19?
2. When did you start to feel ill or experience symptoms?
3. When did you take the COVID-19 test?
4. Were you working from home before you showed symptoms and/or tested positive for COVID-19?
5. Were you using any precautionary measures before you contracted COVID-19? If so, please share what measures you had in place (eg, strict adherence to social distancing, frequent use of masks in public spaces).
6. Do you know how you contracted COVID-19? If yes, please share as much detail as possible regarding how you contracted COVID-19.
7. Do you know of any others who may have contracted COVID-19 from you?

Responses to the above questions were summarized and are reported in a single subsection in the Results section.

### A Deeper Look at COVID-19 Symptoms

Within the full survey, there were a total of 2 questions that addressed topics related to COVID-19 symptoms, including the following:

1. Did you visit a hospital or clinical care for treatment during the course of your illness?
  [If you were hospitalized], how many days were you hospitalized for?
2. What symptoms did you experience [while ill with COVID-19]? Check all that apply.
  [If “other” symptoms is selected from the above checklist] What other symptoms did you experience?

Responses to the above questions were summarized and are reported in a single subsection in the Results section.

### The Road to Recovery From COVID-19

Within the full survey, there were a total of 4 questions that addressed topics related to the road to recovery from COVID-19, including the following:

1. Are you fully recovered from COVID-19?
  [If you still have lingering symptoms], please list the lingering symptoms that you currently have.
2. When do you believe you fully recovered from COVID-19 and associated symptoms?
3. How many weeks did it take you to recover from majority of the symptoms associated with COVID-19?
4. Have you experienced any stigma associated with having COVID-19?
  [If yes], please share more about your experience with stigmas associated with having COVID-19.

Responses to the above questions were summarized and are reported in a single subsection in the Results section.

### Insights From COVID-19 Survivors

Within the full survey, there was 1 open-ended question that addressed topics related to insights from COVID-19 survivors, namely:

Are there any comments or insights that you want to share with regards to your experience with COVID-19?

Responses to the above question were summarized and are reported in a single subsection in the Results section.

## Results

### Onset, Testing, and Contraction of COVID-19

**Figure 1:**
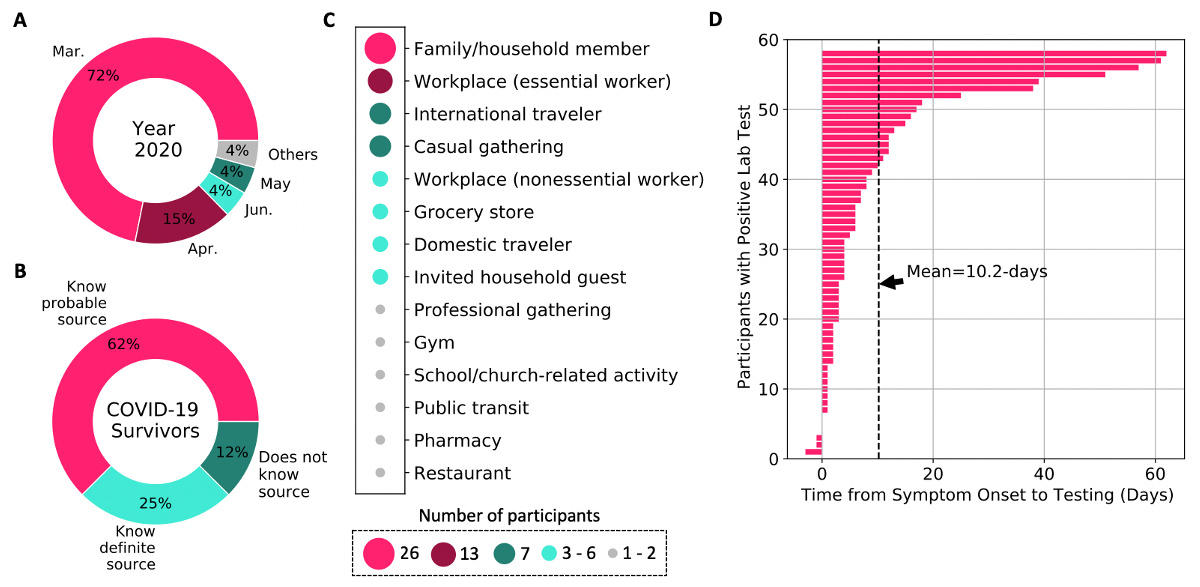
Overview of COVID-19 onset, testing, and contraction for participants in this study. (A) Month of symptom onset; (B) knowledge about the source of infection; (C) prevalent sources of virus transmission; and (D) time lag from symptom onset to testing for COVID-19.

Figure 1A shows that 51 of 72 (72%) participants experienced onset of COVID-19 symptoms in March 2020, about 40 days after the first reported case in the United States [15]. Moreover, 30 of 72 (42%) participants were working from home at the time of their symptom onset, whereas 42 (58%) were not working from home. In addition, 46 of 72 (64%) participants reported to have been practicing the recommended precautionary measures prior to the onset of their symptoms, such as use of masks (n=25), social distancing (n=22), frequent handwashing (n=14), and use of gloves (n=12). However, it is important to note that the majority of participants experienced symptom onset during the first accelerated spread of COVID-19 in the United States, in March 2020, during which recommendations for personal protective practices were only beginning to unfold [6]. In retrospect, 63 of 72 (87%) participants could narrow down the source of how they might have contracted SARS-CoV-2; 62% of these participants shared probable sources, whereas 25% shared what they considered to be definite sources of their infection. From the reported data shown in Figure 1B and 1C, the most prevalent source of virus contraction was from a family or household member. More specifically, 26 of 72 (36%) participants attributed the source of their virus to a family or household member. Additionally, 27 (38%) participants reported that at least one other person in their family or household had contracted COVID-19 from them. This finding suggests that there is a moderate probability of the coronavirus spreading among persons sharing a living space even when the appropriate precautions are taken to minimize the risk. However, emphasis on family or household transmission of COVID-19 is limited. The second most prevalent source of virus transmission was the essential worker who contracted the virus while on the job. This was reported by 13 of 72 (18%) participants. These two sources of virus transmission were also the top sources identified from persons who shared more definite sources of their infection. The results obtained show other sources of virus transmission reported by the participants, such as casual gatherings (7/72, 10%), workplace for nonessential workers (6/72, 8%), and even the grocery store (4/72, 6%).

During the early months of the outbreak in the United States, limitations in testing was identified as one of the multiple factors that contributed to the rapid spread of the disease [6,8]. Figure 1D shows that following infection, participants experienced large variability in the time it took to gain access to COVID-19 testing; this ranged from 3 days before symptom onset to 62 days after symptom onset among this study’s participants. The average time from symptom onset to testing was 10.2 days, with about 10% of the participants getting tested more than 35 days after their symptoms began. Although this study required a COVID-19–positive test to be eligible for participation, we learned during the recruitment phase that a large number of people were continuously denied access to testing during their illness. For example, a patient-led study with over 600 participants who experienced COVID-19 symptoms show that “47.8% were either denied testing or not tested for another reason” [16]. The delay in testing and uncertainty that persons with COVID-19 symptoms experienced is a possible contributor to the further spread of the virus. In addition, a few participants reported that they received treatment for sinus infections, bronchitis, and other conditions without testing, as a result they may not have isolated early in their journey with the virus due to lack of knowledge.

### A Deeper Look at COVID-19 Symptoms

**Figure 2:**
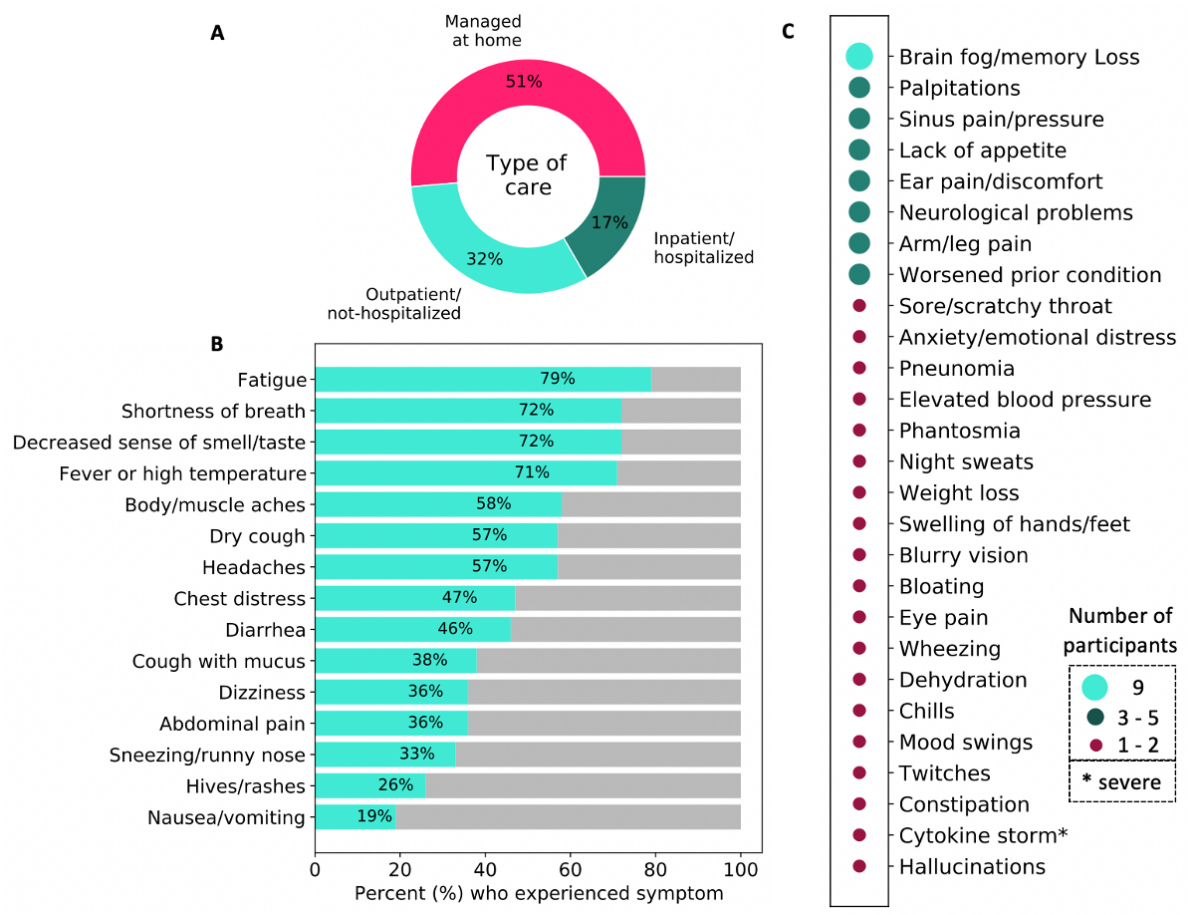
Overview of the type of care received and COVID-19 symptoms reported by participants in this study. (A) The type of care received by COVID-19 survivors; (B) list and prevalence of more common COVID-19 symptoms; and (C) other less-documented symptoms of COVID-19.

There is no dearth on the reported characteristics of COVID-19 for severe cases among hospitalized patients [5,7,17,18]. However, severe cases account for only about 20% of the total population of confirmed COVID-19 cases, and several differences have been identified between mild and severe cases [2,19]. Therefore, it is critical to also understand the journey of nonhospitalized persons with COVID-19 who account for 80% or more of the confirmed cases. The majority of participants in this study (60/72, 83%) managed their symptoms at home and/or in isolation (see Figure 2A); these participants represent people with mild-to-moderate severity of COVID-19. This is in alignment with the estimates in the current literature that about 80% of the COVID-19 cases are mild to moderate as opposed to severe [2,18]. Participants were asked to identify all symptoms they experienced during their illness with COVID-19. Figure 2B shows the prevalence of the well-recognized COVID-19 symptoms reported in this study population (N=72), with the top-4 reported symptoms being fatigue (57/72, 79%), shortness of breath (52/72, 72%), decreased sense of smell or taste (52/72, 72%), and fever (51/72, 71%). These more common symptoms have also been identified in earlier studies, albeit with different percentages for frequency of occurrence in various populations [18,20,21]. More interestingly, in addition to the well-recognized symptoms of COVID-19, 30 of 72 (41%) participants reported other less-documented symptoms that they experienced during their illness. Figure 2C shows a list of other patient-reported symptoms associated with COVID-19 and the prevalence of each symptom among the study population. The most prevalent patient-reported symptoms include brain fog or memory loss, which was reported by 9 (13%) participants; palpitations (ie, elevated heart rate) and sinus pain or pressure, both reported by 5 (7%) participants; and lack of appetite, ear pain or discomfort, and neurological problems, all of which were reported by 4 (6%) participants each. Many of these other symptoms have not been emphasized in prior work. From the list of 27 additional patient-reported symptoms, cytokine storm is considered the most severe and can lead to rapid deterioration, multiple organ failure, and mortality [22-24]. Cytokine storm is characterized by a lethal overreaction of the immune system in response to an infection or disease. The single participant who experienced cytokine storm (male, 62 years) was tested for COVID-19 on April 4, 2020, and then examined by a registered nurse; however, signs of the ensuing havoc were not identified at that point. Following clinical evaluation, the participant was advised to self-quarantine; however, within 72 hours, he was admitted to the hospital as “COVID-19 had triggered a massive cytokine storm.” He was hospitalized for 36 days, on the ventilator for 9 days, and needed surgery to repair the collapse of his left lung due to COVID-19. Fortunately, he lives to tell his story and strongly urges that COVID-19 patients should be evaluated, early in their illness and possibly during testing, for signs of cytokine storm to prevent rapid deterioration and save lives.

### The Road to Recovery From COVID-19

**Figure 3:**
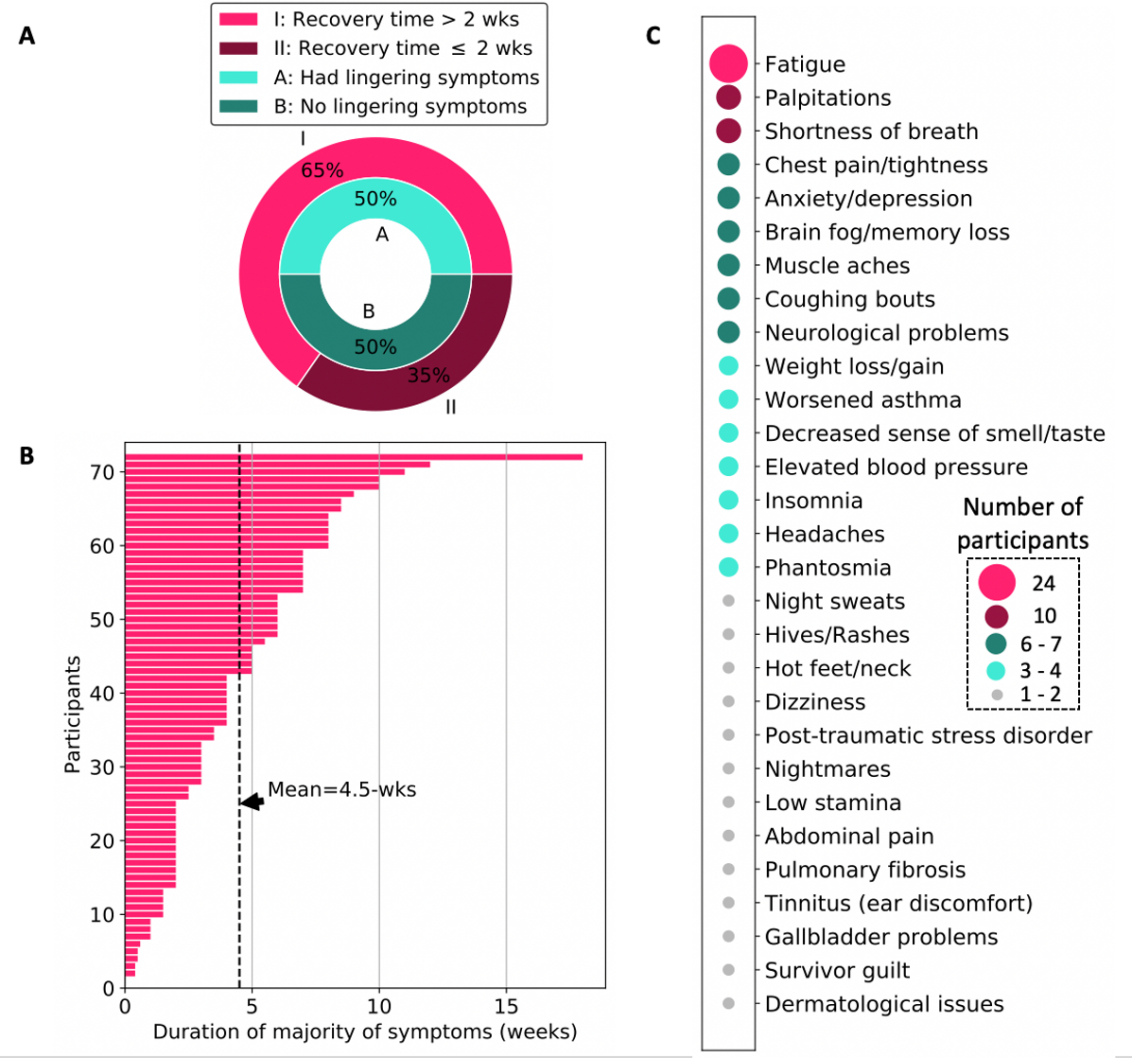
Overview of the road to recovery from COVID-19 for participants in this study. (A) Percentage of COVID-19 survivors who experienced lingering symptoms and a recovery time longer than the commonly cited 2-week period; (B) duration of the majority of symptoms reported (mean duration: 4.5 weeks); and (C) list and prevalence of lingering symptoms associated with COVID-19.

There are many unknowns about the road to recovery for persons who contract COVID-19. According to a February 2020 remark from the World Health Organization (WHO) Director-General, the recovery time is 2 weeks for persons with mild disease and 3-6 weeks for persons with severe or critical disease [25]. However, our results show that 47 of 72 (65%) participants experienced a recovery time longer than 2 weeks (see Figure 3A). More specifically, we found that the mean time to recover from the “majority of symptoms” across all participants was 4.5 weeks; the average recovery time for nonhospitalized patients (ie, patients with mild-to-moderate disease severity) was 4.2 weeks, whereas the average recovery time for hospitalized patients (ie, patients with a more severe experience of COVID-19) was 6.2 weeks or more. A few hospitalized patients reported that they had not recovered from majority of their symptoms at the time of participating in the study; hence, the length of their illness at the time of participation was used as a proxy (ie, date of participation minus date of symptom onset). Our study also shows that COVID-19 survivors experienced high variability in their recovery time from the illness, ranging from 0 days as reported by asymptomatic patients to 18 weeks as reported by a 58-year-old female participant with one pre-existing condition and 3 hospital visits for COVID-19–related symptoms, including dehydration and vestibular migraine.

In addition to the longer recovery times identified in this study, we found that 36 of 72 (50%) participants still experienced lingering symptoms of COVID-19 even after an average of 65 days following their illness onset. A list of such lingering symptoms and changes to general health identified by participants of this study is illustrated in Figure 3C. The most common issues identified include fatigue reported by 24 of 72 (33%) participants; followed by shortness of breath and palpitations reported by 10 (14%) participants; and chest pain or tightness, anxiety or depression, brain fog or memory loss, muscle aches, and coughing bouts—all reported by 6-7 (8%-10%) participants each. As shown in Table 2, 37 of 72 (51%) participants reported having experienced stigma following their contraction of COVID-19, whereas 35 (49%) reported otherwise. There were 9 themes that stood out from further descriptions of the stigmas experienced by those who contracted COVID-19. The most prevalent stigma was avoidance by others after recovery (12/72, 17%), followed by people undermining the illness or experience with COVID-19 (5/72, 7%). Other examples of stigma reported include hostility and dismissal from clinical or medical staff and being blamed for contracting and spreading the virus.

**Table 2:** Experience with stigma reported by participants (ie, COVID-19 survivors) in the C19 Insider Scoop Study (N=72).

Observation		Value, n (%)
**Experienced stigma associated with having COVID-19**		
	Yes	37 (51)
	No	35 (49)
**Examples of stigmas reported**		
	Avoidance by others (eg, friends or neighbors) after recovery	12 (17)
	People undermining the illness or experience with COVID-19	5 (7)
	Hostility and dismissal from clinical or medical staff	4 (6)
	Blame for contracting and spreading the virus	4 (6)
	Shame and name-calling	3 (4)
	Discrimination at places of work and living	3 (4)
	Blame for the stay-at-home orders and the economic impact	1 (1)
	Reprimand from household members at risk of exposure	1 (1)
	Organizations expressing unjust entitlement to medical information	1 (1)

### Insights From COVID-19 Survivors

COVID-19 survivors are an invaluable resource not being fully utilized as a source of knowledge. Through the surveys conducted in this study, these survivors shared insights that they believe will help the society at large better cope with the realities of the ongoing pandemic. Many of these insights are targeted toward the larger population who may not yet have been infected by the coronavirus; however, given the recent rise in COVID-19 cases in the United States and other countries [10], these insights are critical for the general public.

Salient insights obtained from these COVID-19 survivors are listed below (with a few direct quotes from participants):

1. “Any symptom can be the virus so self-isolate even if you have a tickle in your throat…It is easy to mistake a mild case of COVID-19 for a cold or allergies” in the early stages.
2. Understand the “fact that doctors and nurses do not have all the answers” so set your expectations accordingly.
3. The journey with COVID-19 can be scary and lonely, as well as physically, mentally, and emotionally tasking. “You have to advocate for yourself every step of the way.”
4. Young and healthy people without any underlying medical conditions can also have a rough journey with COVID-19. Take it seriously. Protect yourselves and others.
5. “The disease presents differently in all patients. It is not a
  one size fits all
  .” For some it is short-lived, whereas others experience long-term health issues due to COVID-19.
6. If you contract the virus, take it day by day. Be patient with the recovery process as it can be long and volatile. Symptoms and lingering symptoms can come in waves.
7. Do not wait too long to go to the hospital (if needed), as a delay has led to worse outcomes for some. Additionally, some patients have gone to the hospital and been dismissed or discharged prematurely. Trust your instinct as the sole person with first-hand knowledge of your own illness.
8. Do not allow distrust of the health care system to be your excuse for having a worse outcome. Ask questions during clinical care to ensure that you are being prescribed the best treatment for your specific situation.
9. Some medications used in severe cases can cause hallucination and uncanny dreams.
10. Stigma during or after COVID-19 is a reality. Others may have various reactions to people who contracted the virus, especially during the initial phase of returning back to society. Some people may be cruel and unkind with their actions or words.
11. COVID-19 tests and antibody tests can be inaccurate. There are persons who test positive for COVID-19 and negative for antibodies, and vice versa.
12. It is not sufficient for organizations to rely on body temperature as a primary means for determining COVID-19 symptoms. Some survivors never experienced fever, whereas others only experienced fever for a short time (eg, 24 hours).

## Discussion

### Principal Findings

This paper presents a descriptive study that summarizes the experiences of first-hand COVID-19 survivors with a particular focus on how they contracted the virus, the range of symptoms observed, the duration of illness, and their experiences on the road to recovery. Our findings show that the top known source of how people contracted COVID-19 was through a family or household member. Participants also reported a range of symptoms that they experienced during their illness, including up to 27 less-documented symptoms such as brain or memory fog, palpitations, ear pain or discomfort, and neurological problems. Another key finding is that the majority of participants (47/72, 65%) experienced a recovery time longer than the commonly cited 2-week period. The mean recovery time in this study was 4.5 weeks, which was more specifically 4.2 weeks for nonhospitalized patients (ie, mild-to-moderate cases) and 6.2 weeks or more for severe cases (ie, patients who were hospitalized for COVID-19 management). In addition, 51% (37/72) of the participants reported that they experienced stigma associated with having COVID-19. Examples of stigmas shared by our study participants include avoidance by others after recovery and people undermining their illness or experience with COVID-19. Finally, participants shared insights from their personal journey with COVID-19 in the hope to inform and encourage the general public, especially others who may not yet have been infected by the coronavirus.

### Comparison with Prior Work

Majority of the early literature that presented the characteristics of COVID-19 primarily focused on persons with severe cases of the disease or hospitalized patients [5,18,20,21]. This created a critical gap in the literature with regard to the majority of COVID-19 cases (~80%) that are categorized as mild or moderate (ie, nonhospitalized cases). One of the first questions related to the COVID-19 pandemic is how is it spreading among people? Early sources of virus transmission in the United States were primarily from international travelers returning to the country, such as the “First [Reported] Case of 2019 Novel Coronavirus in the United States” [15]. However, community spread became prevalent not too long after. Later reports by the CDC identified transmission through a family member or work colleague as a prevalent source of virus contraction as well as transmission through social gatherings such as dining at a restaurant [26,27]. Results from this study show agreement that family or household- and work-related transmission of COVID-19 is prominent.

Other studies have now begun to uncover characteristics of mild-to-moderate cases of COVID-19. For example, Liu et al [19] reported viral shedding patterns observed in patients with mild and severe COVID-19. They identified some key differences between patients with mild and severe cases of COVID-19, thereby supporting the need for studying both populations comprehensively. Xu et al [28] presented a telemedicine system to continuously monitor the progression of home-quarantined patients with COVID-19, 74 of whom had confirmed infection. They identified symptoms and trajectories that were indicative of the need for hospitalization. More closely related to this work, Jeon et al [29] used a combination of biomedical literature and social media data to characterize the symptoms of COVID-19. They identified 25 novel symptoms by analyzing social media posts, specifically Twitter posts. Some of the less-common symptoms identified in their work align with the less-documented symptoms reported by the participants of this study, such as ear and eye problems, weight loss, and memory disorder. However, this study’s findings also include symptoms that were not identified by Jeon et al [29], such as worsened pre-existing condition (eg, asthma) and elevated blood pressure.

Even fewer studies in the literature have focused on understanding the time to recovery after COVID-19 infection. One of the earliest sources of information on recovery time was a February 2020 remark from the WHO Director-General, which stated an average recovery time of 2 weeks for persons with mild disease and 3-6 weeks for those with severe or critical disease [25]. Subsequently, a CDC study found that approximately 35% of symptomatic outpatients with COVID-19 had not returned to their baseline health 14-21 days after the test date [9]. Carfi et al [30] assessed persistent symptoms in patients after acute COVID-19 and found that 87.4% of patients with severe disease reported at least 1 symptom, particularly fatigue and dyspnea, after an average of 60 days from symptom onset. Results from this study show the average recovery time for mild cases is 4.2 weeks and that for severe cases is 6.2 weeks or more. This finding is consistent with the findings from the CDC report [9] and those reported by Carfi et al [30]; however, our results provide concrete numbers based on participants in this study.

### Limitations

There are several limitations to this study. First, the sample size of the study is small compared to the total number of people in the United States that have contracted COVID-19. However, this study benefits from diverse recruiting strategies and thus has representation from 22 of the 50 states in the country. Second, this study relies on participants’ ability to recall and share their experience with COVID-19. Self-report–based studies have well-known limitations such as recall bias. However, we believe this bias is minimized because the majority of the study participants experienced symptom onset in March 2020 and were recruited between the months of May and June 2020, suggesting that these participants were recruited about 2 months after they contracted the virus. In addition, the average duration of illness was 4.5 weeks across all participants, and participation in this study required recovery from COVID-19–related illness. Hence, we expect that the majority of the participants enrolled into the study within their first month after recovery, thus maximizing their chance to accurately remember their experience.

### Conclusions

This study presents preliminary findings from a small cohort of COVID-19 survivors. It is well known that many people do recover from COVID-19; however, the results from this study show that the journey for some may be long and uncomfortable. To support an accurate depiction of the journey with COVID-19, it is important to recognize that the recovery time can be more than 2 weeks for patients with mild symptoms. In addition, a notable proportion of COVID-19 survivors are considered “long haulers” [31,32]. This refers to people who experience lingering symptoms weeks and even months after initial contraction of the virus. More research is needed to understand the reasons for such diverse experiences with the novel coronavirus. Moreover, thousands of people have died due to infection with the same virus with disproportionate percentages reported among Black and Hispanic populations in the United States [33]. More research is therefore needed to better understand the trajectory of cases that ended in fatality and help prevent a similar outcome for others. One of such efforts should be toward the early detection of the cytokine storm triggered by SARS-CoV-2, as this has been shown to cause fatality for some of the affected population.

## Appendix

Multimedia Appendix 1 Survey questions administered to study participants.

## Abbreviations

CDC: Centers for Disease Control and Prevention
CHERRIES: Checklist for Reporting Results of Internet E-Surveys
CPHS: Committee for Protection of Human Subjects
NIH: National Institutes of Health
WHO: World Health Organization
